# Targeting CDC7 sensitizes resistance melanoma cells to BRAF^V600E^-specific inhibitor by blocking the CDC7/MCM2-7 pathway

**DOI:** 10.1038/s41598-019-50732-w

**Published:** 2019-10-02

**Authors:** Shaimaa A. Gad, Hamdy E. A. Ali, Rofaida Gaballa, Rania M. Abdelsalam, Mourad Zerfaoui, Hamed I. Ali, Salwa H. Salama, Sanaa A. Kenawy, Emad Kandil, Zakaria Y. Abd Elmageed

**Affiliations:** 1 Department of Pharmaceutical Sciences, Rangel College of Pharmacy, Texas A&M Health Science Center, Kingsville, TX 78363 USA; 2 Department of Pharmacology and Toxicology, Faculty of Pharmacy, Cairo University, Giza, Egypt; 3 Department of Surgery, Tulane University School of Medicine, New Orleans, LA 70118 USA; 4 Department of Pharmacology, Medical Research Division, National Research Center, Cairo, Egypt; 5 Department of Biochemistry, Faculty of Pharmacy, Beni-Suef University, Beni-Suef, Egypt

**Keywords:** Cancer, Cancer therapeutic resistance

## Abstract

Although the utilization of selective BRAF^V600E^ inhibitors is associated with improved overall survival in patients with metastatic melanoma, a growing challenge of drug resistance has  emerged. CDC7 has been shown to be overexpressed and associated with poor prognosis in various cancers including melanoma. Thus, we aimed to elucidate the biological role of CDC7 in promoting Vemurafenib resistance and the anticipated benefits of dual targeting of BRAF^V600E^ and CDC7 in melanoma cells. We performed exosomes-associated microRNA profiling and functional assays to determine the role of CDC7 in drug resistance using Vemurafenib-sensitive and resistant melanoma cells. Our results demonstrated that Vemurafenib-resistant cells exhibited a persistent expression of CDC7 in addition to prolonged activity of MCM2 compared to drug-sensitive cells. Reconstitution of miR-3613-3p in resistant cells downregulated CDC7 expression and reduced the number of colonies. Treatment of cells with low concentrations of CDC7 inhibitor TAK-931 sensitized resistant cells to Vemurafenib and reduced the number of cell colonies. Taken together, CDC7 overexpression and downregulation of miR-3613-3p were associated with Vemurafenib resistance in BRAF^V600E^- bearing melanoma cells. Dual targeting of CDC7 and BRAF^V600E^ reduced the development of resistance against Vemurafenib. Further studies are warranted to investigate the clinical effect of targeting CDC7 in metastatic melanoma.

## Introduction

BRAF is a driver oncogene in various human cancers including melanoma, and was the first described oncoprotein with serine/threonine kinase activity^1,2^. Among all identified BRAF missense mutations, BRAF^V600E^ with a single nucleotide transversion from valine to glutamate at position 600 is the most clinically prevalent mutation^3,4^. The mutational activation of BRAF^V600E^ increases BRAF kinase activity extremely higher than the wild-type BRAF, leading to a subsequent 4.6-fold persistent activation of ERK1/2 signaling cascade^5,6^. The mutant BRAF^V600E^ was identified as the bona fide transforming oncogene in malignant melanoma, contributing to 70% of melanoma cases^3^. Due to the potential contribution of mutationally dysregulated kinases in melanomagenesis and progression, BRAF^V600E^ was identified as a promising target for clinically effective therapeutics, including Vemurafenib^7^. Vemurafenib (Zelboraf^®^, PLX4032), is a potent specific inhibitor of BRAF^V600E^, which was the first FDA approved drug against BRAF-mutated metastatic melanoma in 2011^8^. Vemurafenib effectively blocks cell growth, angiogenesis, invasion and metastasis and induce tumor cell death in BRAF^V600E^-associated melanomas^7,9–11^. Despite the remarkable clinical benefits associated with the selective BRAF^V600E^ inhibition by Vemurafenib, emergence of drug resistance hampered the treatment of metastatic melanoma. Approximately 50% of melanoma patients acquire resistance against Vemurafenib, limiting its anti-tumor efficacy and reviving tumor progression within 6 to 8 months of treatment^12^. Accordingly, there is a critical need to understand the molecular mechanisms underlying resistance against BRAF^V600E^-targeted therapy, in an attempt to develop novel targeted therapies that circumvent the challenge of drug resistance in metastatic melanoma and maximize patients’ survival.

In addition to other suggested mechanisms of resistance, acquired resistance to BRAF^V600E^ inhibitors is predominantly linked to MAPK pathway reactivation^9,13^. Interestingly, overexpression of specific microRNAs (miRs) restore the sensitivity of resistant melanoma cells by downregulating their target genes, which are closely related to acquired resistance to BRAF^V600E^ inhibition^14^. This makes miRs as attractive therapeutic targets in resistant melanomas. Due to the genetic complexity of melanomas and the concomitant activation of multiple signaling pathways, combinational therapy has been suggested to enhance the efficacy of treatment^15^.

Cell cycle dysregulation has been shown to mediate resistance of melanoma cells to anti-cancer drugs comprising MAPK inhibitors^10^. One of the most crucial regulator of the cell cycle is the cell division cycle 7 (CDC7)^16^. CDC7 is a highly conserved serine-threonine protein kinase, which is required for initiating the replication machinery. It particularly regulates G1/S phase transition, which is fundamental in cells proliferation, and its deregulation leads to oncogenesis^16^. CDC7 phosphorylates MCM2-7 for the helicase activation by uncoiling the double-stranded DNA as an initial step in DNA replication^17,18^. CDC7 that has been shown to be overexpressed in various cancers^16^ was interrelated with poor prognosis including melanoma^18^. Although various CDC7 pharmacological inhibitors have been developed exhibiting substantial antitumor activity as a single agent, no studies have been conducted on CDC7 inhibition as an alternative targeted therapy in BRAF inhibitors-resistant melanoma cells. Recently, the safety, tolerability, and activity of CDC7-specific inhibitor TAK-931 was evaluated in patients with solid tumors and showed a clinical promise^19^.

In this study, resistant A375-NRAS^Q61K^ (mutant NRAS) and WM983B-BR (wild-type NRAS) melanoma cell lines were used, which harbor BRAF^V600E^, and exhibit resistance to BRAF inhibitor, Vemurafenib, compared to its parental cells. We utilized recent CDC7 inhibitor, TAK-931, as a therapeutic option to circumvent the challenge of developed Vemurafenib resistance in melanoma cells.

## Results

### Differential sensitivity of melanoma cells towards BRAF^V600E^-specific inhibitor Vemurafenib

**Figure 1:**
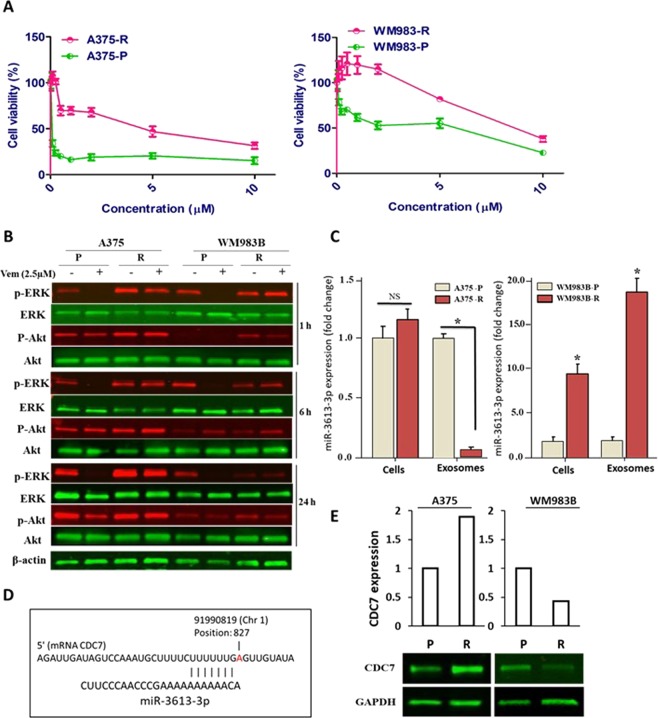
Validation of resistant melanoma cells A375-R and identification of miR-3613-3p as a potential target of CDC7. (**A**) Cytotoxic effect of Vemurafenib was assessed on two parental (P) and two resistant (R) melanoma cells after treating the four cells with various concentrations of Vemurafenib ranging from 0.1 to 10 µM for 5 days. Each experiment performed in triplicates and independently repeated three times. (**B)** Western blot analysis was performed to examine the activity of ERK1/2 and Akt in parental A375-P and WM983B-P and their corresponding resistant cells after 2.5 µM Vemurafenib treatment at different time points. β-actin was used as a loading control protein. (**C**) qPCR analysis was performed to validate the expression of miR-3613-3p in exosomes and cell lysate of A375-P and WM983B-P regarding their corresponding resistant cells using RNU6 and 5 s rRNA as internal controls. (**D**) Predication of CDC7 as a target gene of mir-3613-3p using available miRmap software (https://mirmap.ezlab.org/app). (**E**) The endogenous expression of CDC7 in the four melanoma cells were assessed by Western blot analysis. Fold change of miR-expression was calculated relative to A735-P & WM983B-P cells. *Depicts significance at p < 0.05.

To distinguish the resistant phenotype of melanoma cells against selective inhibition of BRAF^V600E^ by Vemurafenib, parental A375 (A375-P) & WM983B (WM983B-P), and resistant A375- NRAS^Q61K^ (A375-R) & WM983B-BR (WM983B-R) were used in this study. As initial step, cell viability assay was performed to assess the differential sensitivity of melanoma cells to Vemurafenib treatment. Figure 1A showed that the concentrations of 0.1 and 2.5 µM Vemurafenib were enough to cause 50% inhibition of cell proliferation in A375-P and WM983B-P, respectively. In contrast, A375-R and WM983B-R cells were insensitive to the increase of Vemurafenib concentrations up to 2.5 and 5 µM, respectively. To further characterize the differential response of the parental versus resistant melanoma cells, the effect of Vemurafenib on MAPK signaling pathway was evaluated by Western blot analysis at different time intervals. As shown in Fig. 1B and Supplementary Fig. 1, the basal level of phospho-ERK1/2 (p-ERK1/2) was detected in both parental and resistant non-treated melanoma cells. However, ERK1/2 activity in A375-P and WM983B-P cells showed a complete inhibition at all selected time points compared to a persistent ERK1/2 activation in A375-R and WM983B-R cells over the course of 24 h treatment. In addition, phosphorylated Akt (p-Akt) showed a reduction in its activity after 24 h of treatment in A375-P and WM983B-P parental versus resistant A375-R and WM983B-R cells.

### miR-3613-3p is downregulated in A375-R cells-associated exosomes and targets CDC7 expression

**Figure 2:**
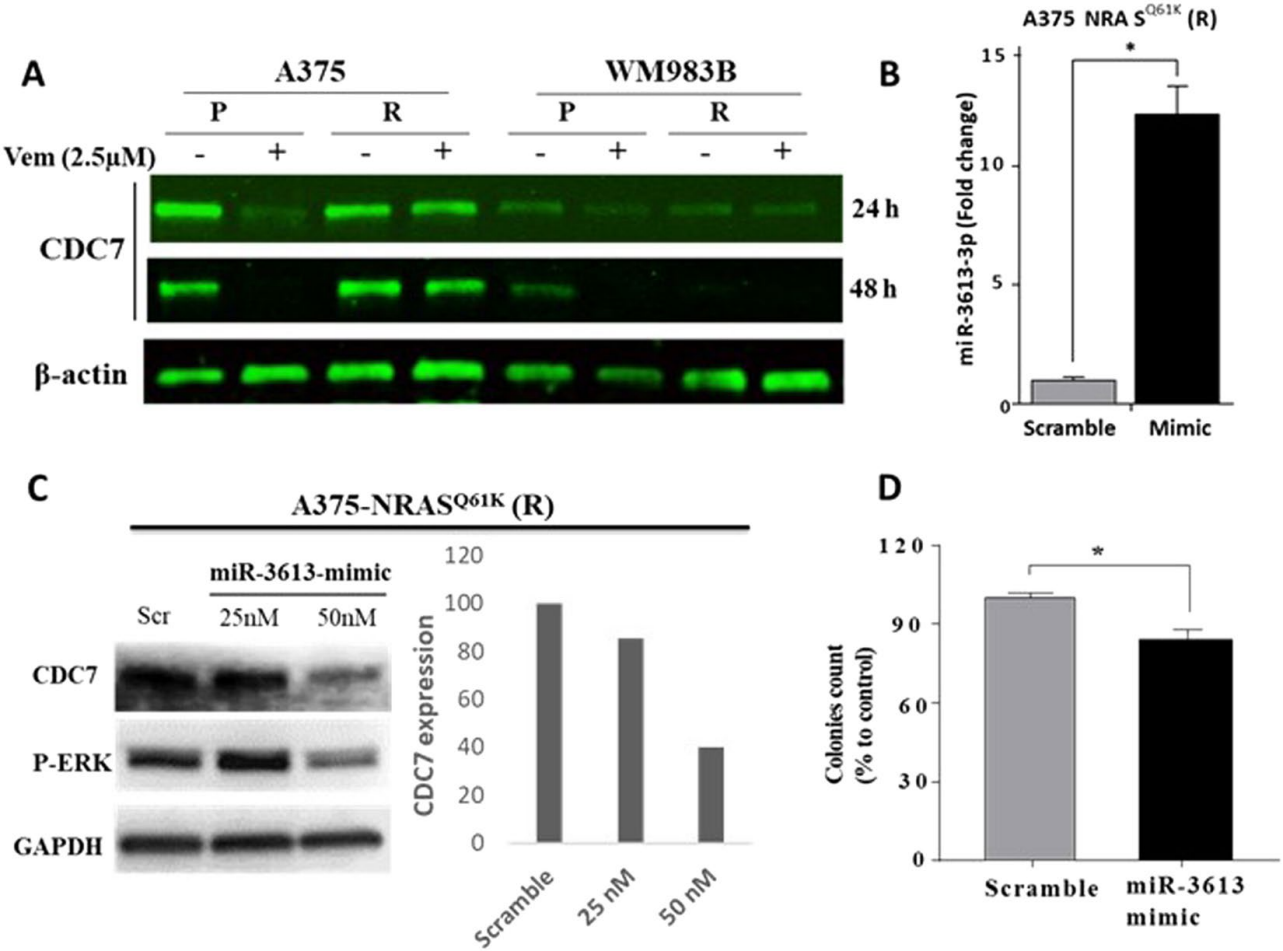
Reconstitution of miR-3613-3p suppresses CDC7 expression and reduces the number of colony formation in A375-R cells. (**A**) Endogenous expression of CDC7 was evaluated in presence and absence of 2.5 µM Vemurafenib after 24 and 48 h by Western blot analysis in parental (A375-P & WM983B-P) and resistant (A375-R & WM983B-R) cells. (**B**) A375-R was transfected with 50 nM miR-3613-3p mimic for 36 h, RNA was extracted and qPCR was performed. (**C**) Cell lysate was collected from the transfected cells with miR-3613-3p mimic (25 and 50 nM) and Western blot analysis was carried out using anti-CDC7 and anti-p-ERK1/2 antibodies. (**D**) Transfected and scrambled cells were cultured in 6-well plates for colony formation assay and kept for 10 days. After staining, colonies were counted and expressed as a percentage to control cells. *Depicts significance at p < 0.05.

We did microarray analysis for exosomes isolated from the conditioned media procured from A375-R regarding its parental cells and we identified a number of up- and down-regulated miRs (Table 1). miR-3613-3p was identified as one of top listed downregulated miRs (~8.5 folds). It has been reported that exosomes contribute to tumor progression, metastasis and drug resistance via transferring fully functional cargo including miRs into recipient cells in tumor microenvironment^20^. Therefore, we isolated miRs from exosomes and from cells to examine whether exosomes can differentially upload miRs in their cargo to promote drug resistance. Next, we validated the expression of miR-3613-3p in the 2 pairs of parental and resistant (A375 & WM983B) melanoma exosomes in addition to their respected cells using Real-Time PCR analysis (Fig. 1C). There was no statistical difference between A375-P and –R on cellular level (p = 0.126); however, a significant downregulation (−6.8-fold, p = 0.0003) was recorded for miR-3613-3p in A375-R exosomes. However, miR-3613-3p was upregulated (p < 0.001) on exosomal and cellular levels of WM983B-R by ~9.5 folds and ~19.5 folds, respectively. Using bioinformatic analyses, we identified several targets for miR-3613-3p; and CDC7 was one of these candidates (Fig. 1D). Obviously, this step was followed by examining the expression of CDC7 in A375 and WM983B cells. Surprisingly, the endogenous expression of CDC7 was higher in resistant versus parental cells in case of A375 cells while the signal of CDC7 was lower in parental and completely lost in resistant WM983B-R cells (Fig. 1E & Supplementary Fig. 2,A). Treatment of cells over 48 h with 2.5 µM Vemurafenib inhibited the expression of CDC7 in parental cells, compared to persistent expression in resistant cells (Fig. 2A & Supplementary Fig. 2B). Since WM983B-R cells have very low expression of CDC7, we utilized A735 cells in the rest of our experiments. To examine the role of miR-3613-3p in the context of drug resistance in melanoma, resistant cells were transfected with either miR-3613-3p mimic at 25 and 50 nM concentrations or scrambled siRNA as a negative control and the success of transfection was validated by qPCR analysis (Fig. 2B). Approximately 48 h post-transfection, protein lysates were collected and Western blot analysis was conducted. As shown in Fig. 2C and Supplementary Fig. 3, reconstitution of miR-3613-3p in A375-R cells showed a slight decrease in CDC7 expression at 25 nM of the miR mimic and reached the maximum inhibition (62%) at 50 nM concentration. While miR-3613-3p overexpressed in A375-R using 50 nM mimic concentration, the activity of ERK1/2 was reduced compared to the control cells. Cell proliferation of transfected cells was assessed by clonogenic assay to determine the effect of miR-3613-3p in A375-R cells. Twenty-four hour after a single transfection of cells with 50 nM mimic, cells were plated at low density and monitored over 10 days. The results of long-term growth assay indicated that overexpression of miR-3613-3p caused colony growth arrest in A375-R cells by 21% (P < 0.01) as compared to scrambled cells (Fig. 2D).

**Table 1:** Differential signature of exosomes-associated miRs in Vemurafenib-resistant versus sensitive melanoma cells.

No.	has-miR	Log2 fold change	Regulation
1	**hsa-miR-302d-3p**	3.6701	Up
2	**hsa-miR-1**	2.0549	Up
3	**hsa-miR-590-5p**	1.6031	Up
4	**hsa-miR-365**	1.0605	Up
5	**hsa-miR-149-3p**	8.594	Down
6	**hsa-miR-3613-3p**	8.453	Down
7	**hsa-miR-664**	8.060	Down
8	**has-miR-25-3p**	7.888	Down
9	**hsa-miR-625-3p**	7.195	Down
10	**hsa-miR-620**	6.990	Down
11	**hsa-miR-208a**	6.453	Down
12	**hsa-miR-4312**	6.259	Down
13	**hsa-miR-3679-3p**	6.016	Down
14	**hsa-miR-675-3p**	5.591	Down
15	**hsa-miR-4268**	5.565	Down
16	**hsa-miR-let-7e**	5.024	Down
17	**hsa-miR-3675-3p**	3.874	Down
18	**hsa-miR-9-3p**	3.651	Down
19	**hsa-miR-214**	2.969	Down
20	**hsa-miR-553**	2.823	Down
21	**hsa-miR-934**	1.951	Down
22	**hsa-miR-1280**	1.490	Down
23	**hsa-miR-488**	1.206	Down
24	**hsa-miR-520a-5p**	1.193	Down

Exosomes were isolated from the conditioned media of melanoma cells and RNA was extracted. miRNA profiling was performed as described in the method section. Up- and downregulated miRs in exosomes of resistant A375-R were expressed as log2 fold change compared to parental A375-P melanoma cells.

### Pharmacological inhibition of CDC7 by its specific inhibitor TAK-931

**Figure 3:**
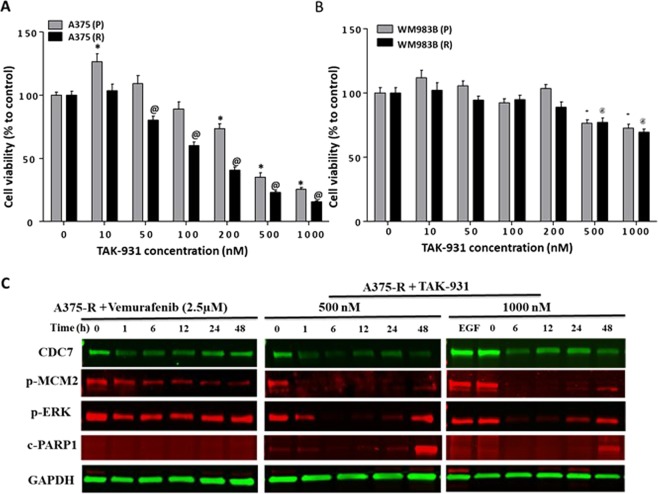
Inhibiting CDC7 by TAK-931 suppressed cell growth of A375 cells. (**A,B**) A375-P & WM983B-P and resistant A375-R & WM983B-R cells were treated for 5 days with different concentrations of TAK-931 and the cytotoxicity was assessed by cell counting kit-8 assay. (**C**) Western blot analysis was performed to compare the expression of CDC7 and the activity of p-MCM2 and p-ERK in A375-R cells after treatment of these cells with 2.5 µM of Vemurafenib and 500 and 1000 nM of TAK-931 for 48 hours. *, ^@^Depicts significance at p < 0.05 relative to untreated parental (P) and resistant (R) cells, respectively.

In an attempt to further understand the contribution of CDC7 to Vemurafenib resistance, CDC7 expression was pharmacologically inhibited by selective CDC7 inhibitor, TAK-931. Cell viability assay was performed to assess the differential sensitivity of melanoma cells to TAK-931. Accordingly, cells were exposed to increasing concentrations of TAK-931 ranging from 10 to 1000 nM for 5 consecutive days. The results revealed that A375-P cells and A375-R showed a concentration-dependent proliferation inhibition when compared to non-treated cells. However, the IC_50_ for TAK-931 was recorded at 150 nM in A375-R and 350 nM in A375-P (Fig. 3A). WM983B cells showed a lower response to TAK-931 compared to A375 cells because WM983-R cells have very low expression of CDC7 (Fig. 3B). Accordingly, we aimed to investigate whether the suppression of CDC7 could be an alternative therapy for resistant melanoma cells to overcome Vemurafenib resistance. Time course of drug treatment up to 48 h was performed for A375-R cells with either Vemurafenib (2.5 µM) or TAK-931 (500 and 1000 nM), and the difference of CDC7 expression and MCM2/ERK1/2 activities were assessed. As expected, CDC7 expression, and p-MCM-2 and p-ERK were persisitant after treating cells with Vemurafenib (Fig. 3C & Supplementary Fig. 4). Remarkably, treating A375-R cells with TAK-931 resulted in inhibition of CDC7 expression as early as 1 h after treaement and this effect was lasting to 24 h. In parallel, p-MCM-2 showed dramatic decrease as early as 1 h up to 48 h after TAK-931 treatment. Moreover, TAK-931 inhibited ERK1/2 activity starting from 6 h up 24 h of treatment at 500 nM concentration. Notably, ceaved PARP1 was mostly recogized after 48 h of treatment (Fig. 3C).

### Inhibition of CDC7 by TAK-931 sensitizes A375-R to Vemurafenib treatment

**Figure 4:**
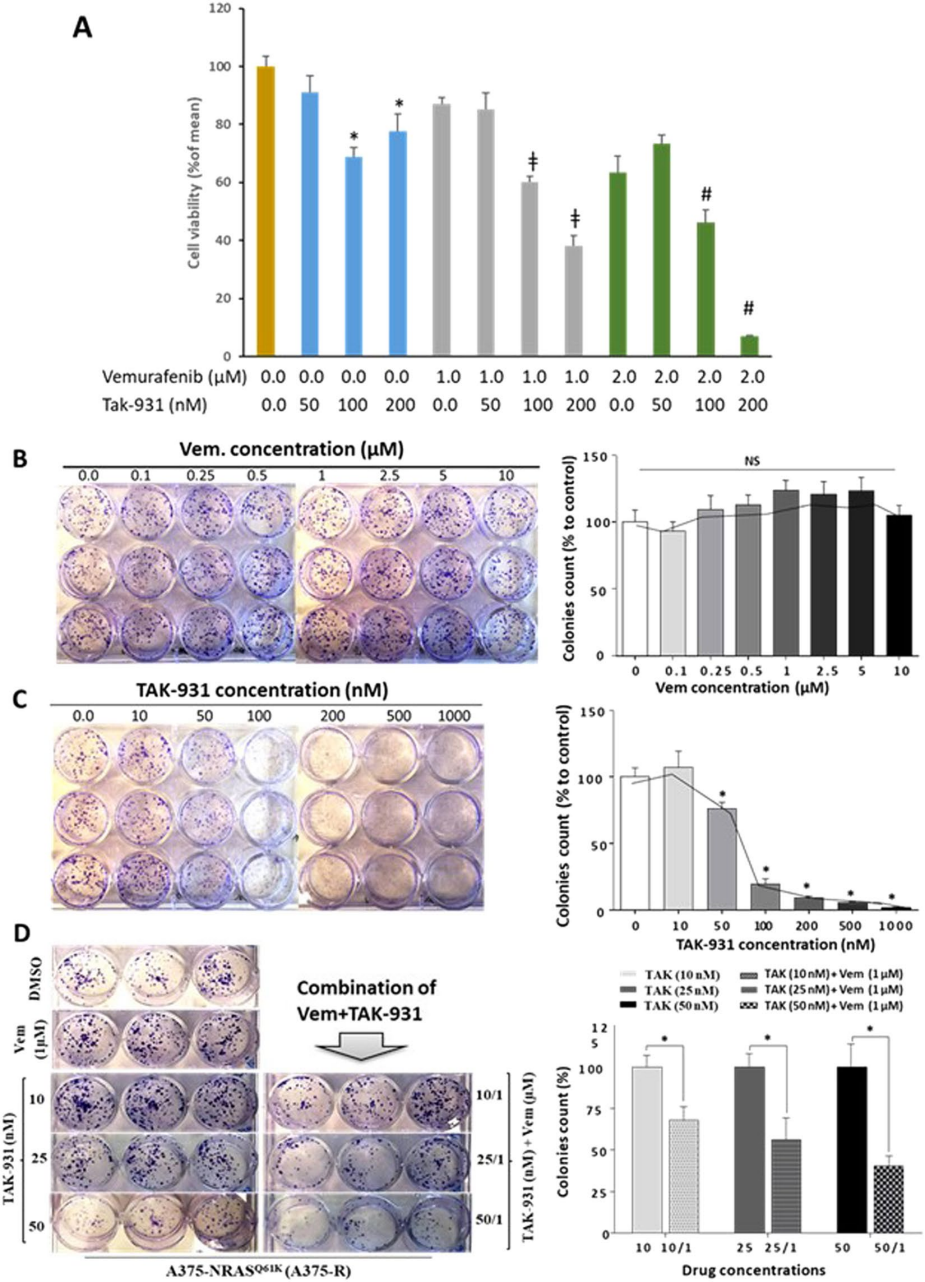
Treatment of A375-R cells with CDC7 inhibitor TAK-931 sensitizes resistant melanoma cells to Vemurafenib. (**A**) Cell proliferation assay for A375-R cells treated with either Vemurafenib (1.0 & 2.0 µM) or TAK-931 (50, 100 & 200 nM) or their combinations for 5 days. Cell cytotoxicity was assessed as indicated and expressed as a percentage of change. (**B,C**) Resistant cells were treated with increasing concentrations of Vemurafenib from 0 to 10 µM or TAK-931 inhibitor ranging from 0.0 to 1000 nM and monitored for 10 days. (**D**) Resistant cells were treated with low concentrations of TAK-931 (10, 25 and 50 nM), in the presence or absence of 1 µM Vemurafenib. The number of colonies was counted per each treatment as indicated and the statistical analysis was performed using one way ANOVA. Each bar represents the mean (%) ± SE of three replicates. NS: non-significant data. *Depicts significance at p < 0.05 relative to control cells (**A–C**) or to those cells individually treated with TAK-931 (**D**). ^ǂ,#^Depicts significance relative to cells treated with 1 µM and 2 µM of Vemurafenib, respectively.

Cell proliferation assay was performed on A375-R cells using two concentrations of Vemurafenib (1.0 & 2.0 µM) or three concentrations of TAK-931 (50, 100 & 200 nM) or the combined drugs. The results showed that the best concentrations caused a significant reduction in cell proliferation were the combination of Vemurafenib (2 µM) with 100 & 200 nM TAK-931 (Fig. 4A). In addition, clonogenic assay was performed to assess the effect of CDC7 inhibition over a longer time period. Resistant cells were plated at low density then exposed to increasing concentrations of either Vemurafenib (0.1 to 10 µM), or TAK-931 (10 to 1000 nM), and monitored for 10 days. The treatment of A375-R cells with Vemurafenib did not effectively inhibit their ability to colonize, and cells exhibited persistent colony growth even at higher concentrations of Vemurafenib (Fig. 4B). Conversely, treatment of A375-R cells with TAK-931 demonstrated a concentration-dependent growth arrest by ~25%, when cells were treated with 50 nM of the drug. Moreover, the number of colonies dropped sharply by 80% decrease, when cells were treated with 100 nM of TAK-931 (Fig. 4C). A375-R cells treated with three different concentrations of TAK-931 in addition to 1 µM Vemurafenib (referred 10/1, 25/1 and 50/1), resulted in more effective TAK-931 concentration-dependent decrease in number of colonies compared to cells treated with individual inhibitors (Fig. 4D). Co-treatment of cells with TAK-931 (10 nM) and 1 µM Vemurafenib (10/1) caused a 32% decrease in number of colonies, while 25/1 and 50/1 combinations further reduced the number of colonies by 44% and 60%, respectively, compared to individual drug treatments as depicted in Fig. 4D.

### Protein expression of CDC7 and its correlation with clinical outcomes in melanoma tissues

**Figure 5:**
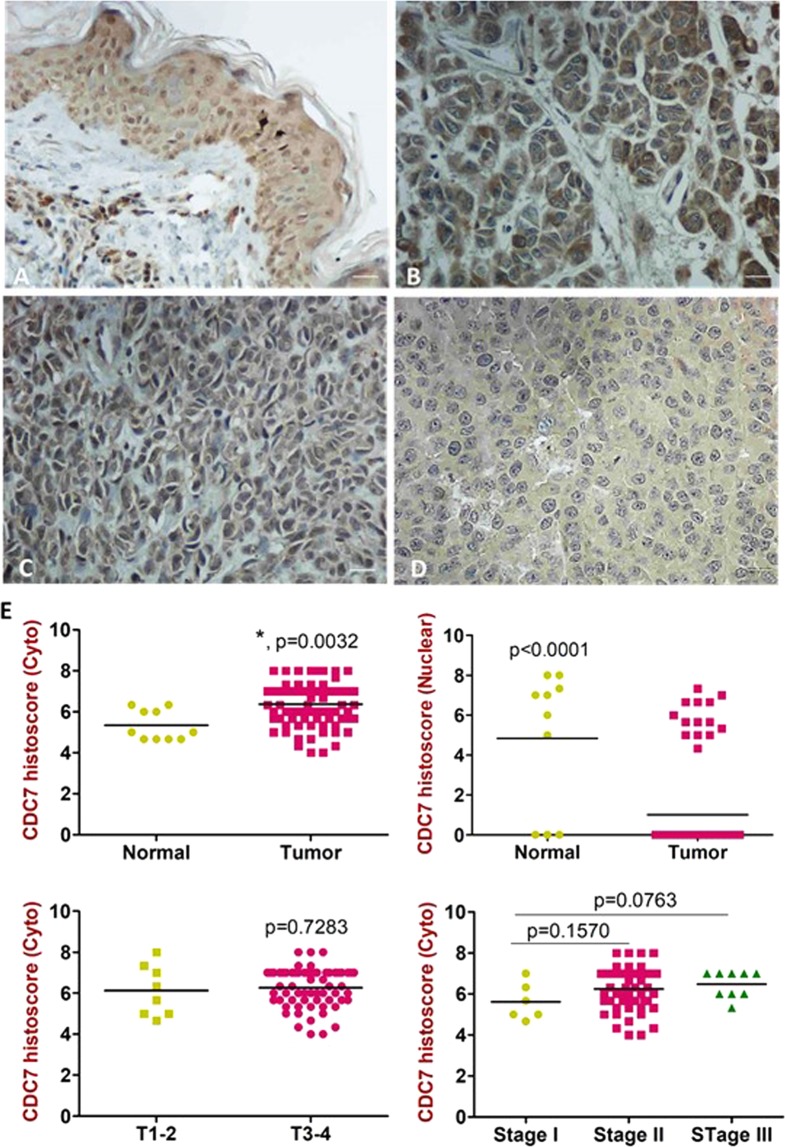
Differential expression of CDC7 in melanoma tissues. (**A–D)** Immunostaining was performed on 100 melanoma tissue cores. Cytoplasmic staining of CDC7 (**B**) was observed in malignant tissues along with other nuclear staining (**A,C**) detected in melanoma and normal skin cores versus very weak staining in other melanoma tissues (**D**). (**E**) IHC score of CDC7 in melanoma tissues versus normal skin for both cytoplasmic and nuclear staining. *Depicts significance at p < 0.05. Scale bar is 200 µm.

To validate the *in vitro* studies of overexpression of CDC7 in human melanoma tissues, we performed IHC on melanoma and normal skin tissues. The immunohistochemical score of cytoplasmic CDC7 was high in 39/92 (42.4%), moderate in 43/92 (46.7%) and low in 10/92 (10.9%) of melanoma specimens. Nuclear staining was observed in 15/92 (16.3%) of melanoma tissues and in 10/10 (100%) of normal skin tissues (Fig. 5). The cytosolic staining of CDC7 in melanoma tissues was higher (p = 0.0032) compared to normal skin tissues and had a trend of significance between Stage I and III (p = 0.0763, Fig. 5E). Correlation studies showed that the cytoplasmic expression of CDC7 was significantly associated with age (r = 0.3195, p = 0.0034), gender (r = 0.2547, p = 0.0209) and pathological stage (r = 0.2810, p = 0.0167). Basically, nuclear staining of the protein was only correlated with pathological stage as shown in Supplementary Table 2.

## Discussion

Although the relative success of melanoma treatment, the emergence of drug resistance still a challenge. To further study the underlying mechanisms contribute to the acquired resistance to Vemurafenib, we utilized Vemurafenib-sensitive A375 & WM983B (-P) and resistant melanoma cells A375-NRAS^Q61K^ and WM983B-BR (-R) cells. Initially, we confirmed that the Vemurafenib resistant melanoma cells kept the acquired resistance phenotype as previously reported^21–23^. In cell based-assay, A375-R & WM983B-R cells treated with Vemurafenib showed a little inhibition in cellular proliferation rate, a case accompanied by consistent hyper-activation of ERK1/2 and Akt activities compared to their respective parental cells. The RAS/RAF active mutations have been detected in cutaneous melanoma and, therefore, suggesting their oncogenic activity in RAS/RAF/MEK/ERK pathway^9,15^. The gain of function of NRAS^Q61K^ mutation constitutively hyper-activates ERK1/2^24^. Melanoma cells bearing secondary NRAS^Q61K^ mutation are more vunerable to dvelop Vemurafenib resistance than cells with primeray BRAF mutations. This evidenced by the fact that the coexistence of NRAS^Q61K^ and BRAF^V600E^ in melanoma cells is sufficient to by-pass Vemurafenib inhibitory effects on ERK1/2 signaling^22^. In addition to other mechanisms, a typical mechanis of resistance is mediated by ERK1/2 hyperactivation in melanoma cells including amplification of BRAF expression, and/or mutational activation of MEK^13^.

Our findings demonstrate that miR-3613-3p was among the most downregulated microRNAs in resistant versus parental A375-derived exosomes. However, restoration of this miR in resistant cells reversed their resistant phenotype and re-sensitized resistant melanoma cells to Vemurafenib as corroborated by our results and other previous studies conducted on resistant melanoma cells using different miRs^14,25^. Although miR-3613-3p has been reported to be dysregulated in various types of cancer, our study provides the first evidence that dysregulation of miR-3613-3p was associated with Vemurafenib resistance in melanoma cells. Prior studies elaborated on the role of miR-3613-3p in the development of drug resistance where it was downregulated in chemoresistant epithelial ovarian cancer cells to paclitaxel and carboplatin treatment^26^, and in resistant breast cancer-derived exosomes^27^.

To identify target gene candidates of miR-3613-3p, bioinformatic analyses predicted that cell division cycle 7 (CDC7) is a potential target for miR-3613-3p and, perhaps, suggesting its role in Vemurafenib resistance^28^. Our results also demonstrate that the endogenous expression of CDC7 was higher in A375-R compared to A375-P cells, whereas the candidate protein was lower or completely absent in WM983B-R cells. This also explained the reason behind downregulation of miR-3613-3p in A375-R versus WM983B-R cells corresponding to their parental control cells. CDC7 is a serine-threonine protein kinase that controls cell cycle progression through its downstream effector minichromosome maintenance 2–7 (MCM2-7), which is crucial player to unwind the double-stranded DNA as an initial step of DNA replication^29^. The DNA replication initiation machinery is a potent regulator of the proliferative state in normal cells and its dysregulation contributes to uncontrolled growth rate in cancer cells^16^. A mounting evidence indicts the overexpression of CDC7 in tumor cells and its relation with the increase in mutational frequency and therefore promotes tumorigenesis and chemoresistance^30,31^. Herein, we report that CDC7 was overexpressed in the cytoplasm of melanoma cells compared to normal skin and its expression was correlated with age, gender and tumor staging. Other previous studies also reported the overexpression of CDC7 in primary and metastatic melanoma compared to benign nevi, and was associated with lower relapse-free survival^18,32^. In the same stream, its notable expression was associated with cell cycle dysregulation, which mediates resistance to MAPK inhibitors in melanoma cells^10^.

In the present study, we found an association between lower expression of miR-3613-3p and ERK1/2 and CDC7 hyperactivation in resistant melanoma cells. Accordingly, reconstitution of miR-3613-3p in resistant A375-R cells using the miR-3613-3p mimic was associated with inhibition of ERK1/2 activity and CDC7 expression. It was also associated with a decrease in number of cell colonies compared to scrambled cells. In a recent study conducted by Li *et al*., the activation of ERK1/2 by EGF activates CK2α to phosphorylate PGK1 and increases its interaction with CDC7 to promote DNA replication^33^. In another study, knockdown of CDC7 in oral squamous cell carcinoma reduced MCM2 and ERK1/2 phosphorylation but promoted Akt phosphorylation^34^. This suggests the link between CDC7 inhibition and reduced ERK1/2 activity. To further understand the link between CDC7 and MAPK pathway in Vemurafenib resistance melanoma, we tested the effect of Vemurafenib treatment on CDC7 expression in parental and resistant melanoma cells harboring BRAF^V600E^. Treatment of cells with Vemurafenib precluded CDC7 expression and ERK1/2 activity in parental, but not in resistant melanoma cells. This implies that CDC7 and MAPK pathway are interrelated, and CDC7 may be affected by Vemurafenib treatment in melanoma cells. However, more studies are needed to closely examine the interconnection between CDC7 and MAPK molecular components.

Inhibition of ERK1/2 activity by Vemurafenib was shown to be associated with reduced cell growth and G1-phase cell cycle arrest, which is mediated by the accumulation of CDK inhibitors^35,36^. A number of studies showed that CDKs essentially interact with CDC7 kinase to eventually activate MCM2-7 complex and promote cell proliferation^16,37^. Similar to CDKs, CDC7 may have a regulatory role on ERK1/2 activity in melanoma cells, which has not been established yet. Based on our findings, BRAF^V600E^ inhibition blocks CDC7-downstream MCM2 and ERK1/2 activities in parental, but not in resistant cells. This was supported by the evidence that Vemurafenib treatment provoked cell cycle arrest associated with cytostatic and cytotoxic effects in parental, but not in resistant cells^38,39^. To further examine whether CDC7 inhibition could sensitize resistant melanoma cells to Vemurafenib, a pharmacologic selective CDC7 inhibitor, TAK-931, was used to manipulate the kinase activity. CDC7 inhibitors have a major potential as new generation of anti-cancer targeted therapies. Inhibition of CDC7 kinase activities resulted in cancer cell-specific apoptosis as a consequence of abrogation of cell cycle progression^30,40^. However, no previous studies have been conducted on CDC7 inhibition as an alternative targeted therapy in Vemurafenib-resistant melanoma cells. Recently, TAK-931 has been introduced as a potent selective ATP-competitive inhibitor for CDC7 kinase. TAK-931 is still under clinical trials after exerting antiproliferative effect using preclinical models. This effect was associated with a dose-dependent suppression of MCM2 phosphorylation in various solid tumors, but not in melanoma^41^. Accordingly, we established a concentration-dependent proliferation inhibition profile of Vemurafenib-sensitive and resistant melanoma cells after TAK-931 treatment. Interestingly, A375-R cells were more sensitive to TAK-931 treatment than A375-P cells. This suggests the biological role of CDC7 in developing resistance in CDC7-positive melanoma cells, and its inhibition could be a potential alternative therapy in Vemurafenib resistant melanoma cells. In contrast, WM983B-R resistant cells did not show the same effect of the drug because they do not express CDC7 as A375-R cells do. Mechanistically, we examined the effect of CDC7 inhibition by TAK-931 treatment on MAPK pathway and CDC7 downstream effector proteins, ERK1/2 and MCM-2 in A375-R cells.

**Figure 6:**
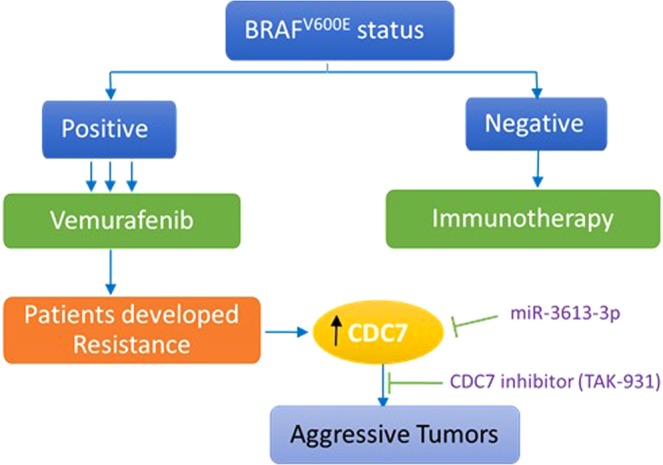
Suggested treatment strategy in Vemurafenib-resistant melanoma patients with CDC7 inhibitor, TAK-931. Diagrammatic representation of the suggested strategy for treatment of melanoma patients with CDC7 inhibitor who had BRAF mutation and developed resistance against Vemurafenib. If CDC7 expression is high in the tissues of these patients, they are expecting to receive CDC7 specific inhibitor TAK-931 sequentially to Vemurafenib treatment and after developing signs of drug resistance. Another strategy is to increase the bioavailability of miR-3613-3p to the tumor cells, where it targets CDC7 and its downstream signaling. This intervention is anticipated to reduce the drug resistance and improve the clinical outcomes of the melanoma patients.

A growing body of evidence shows that targeting additional signaling cascades provides additional benefit as anticancer therapy^11^. A recent study showed that mutant NRAS melanoma cells better respond to combinational therapies with better outcomes^42^. Taking the same approach, our results showed that the combination of the two drugs; Vemurafenib and TAK-931, inhibited the growth of melanoma cells compared to monotherapy. This approach may block other compensatory mechanisms involved in NRAS-dependent resistance^22^. Another advantage of using low concentrations of the two drugs is to minimize the side effects raised from the option of using monotherapy. The current strategy of melanoma treatment mainly depends on the molecular feature of tissue specimens. This study may offer a new treatment option for those patients with BRAF^*V600E*^-positive and developed resistance against Vemurafenib and have higher CDC7 expression to receive TAK-931 inhibitor in combination with the offered drug as illustrated in Fig. 6. Further studies are warranted to examine the effect of TAK-931 and Vemurafenib combination using preclinical model as a second step for reducing drug resistance in melanoma patients. In addition, melanoma specimens procured from patients with vemurafenib resistance will assist to better understand the role of CDC7 in drug resistance.

## Materials and Methods

### Cell culture

All experimental methods and protocols included in this study were performed in accordance with the National Institute of Health (NIH) and Research Compliance and Biosafety guidelines approved by Texas A&M University (IBC permit: 2016-163), College Station, Texas. Vemurafenib-resistant A375-NRAS^Q61K^ (A375-R) and its parental A375 (A375-P) cells, established from 54-y old female malignant melanoma patient, were kindly provided by Dr. Andrew E. Aplin (Thomas Jefferson University, Philadelphia, Pennsylvania). Parental metastatic cells WM983B (WM983-P) established from inguinal node of 54-y old male and matched resistant WM983B-BR (WM983-R) cells were received from Dr. M Herlyn (Wistar Institute, Philadelphia, PA). WM983B cells harbor mutant BRAF^V600E^, and wild-type for NRAS, c-kit and CDK4. A375 cells were grown in DMEM (Dulbecco’s Modification of Eagle’s Medium) containing 4.5 g/L glucose, L-glutamine and sodium pyruvate, 10% FBS. WM983B cells were cultured in MCDB153 medium (Sigma-Aldrich, St. Louis, MO), containing 20% Leibovitz L-15 medium, 2% FBS, 5 µg/ml insulin. Cells were grown in1% penicillin/streptomycin and maintained at 37 °C in a 5% CO_2_ atmosphere.

### Isolation of exosomes and miR profiling

Exosomes were isolated from the conditioned media of A375-P & -R cells by ultracentrifugation as previously described^43^. RNA was extracted from the exosomes using TRIzol method following the standard protocol (ThermoFisher Scientific, Waltham, MA). The purified RNA (1 µg each) was subjected to miR profiling using miRCURY LNA™ microRNA array kit (Qiagen, Germantown, MD), as reported^43^.

### Cytotoxicity assay

About 2 × 10^3^ cells were seeded in 96-well plate by adding 100 µl of the prepared cell suspension per each well and kept to settle down overnight. Next, the old media were aspired and cells were treated with different Vemurafenib (PLX4032) (Selleck Chemicals LLC, Houston, TX) concentrations ranging from 0.1 to 10 µM in triplicates, and incubated for 5 consecutive days with replenishment of the drug every 2 days. DMSO (0.1%) was used as a negative control. Cell toxicity was evaluated using cell counting kit-8 following the standard protocol (Dojindo Molecular Tech. Inc., Rockville, MD). The developed color was measured by microplate reader at 450 nm (AccuSkan FC plate reader, Thermo Fisher Scientific, Waltham, MA).

### Clonogenic assay

Three hundred cells suspended in 1 ml of culture media were seeded per well in 12-well plate. Next day, cells were treated in triplicates with different concentrations of either Vemurafenib or CDC7 specific inhibitor TAK-931 (Chemietek, Indianapolis, IN), and 0.1% DMSO served as a control. Each drug was replaced with freshly prepared one every other day. After ending of incubation period, the generated colonies were thoroughly washed with PBS, and followed by 4% paraformaldehyde fixation for 15 min at room temperature. The paraformaldehyde was then aspirated and the fixed colonies were stained with 0.5% crystal violet for 25 min. Plates were then washed with water and left overnight to dry out before imaging and counting.

### Transfection of melanoma cells with microRNA mimics, RNA extraction and Real-Time PCR (qPCR) analysis

Melanoma cells were cultured in 12 well-plate and about 70% confluent cells were transfected with miR-3613-3p and All Stars negative siRNA AF488 as a negative control using HiPerFect transfecting reagent (Qiagen Inc., Germantown, MD). Forty eight hours later, total RNA was extracted from melanoma cells using Trizol reagent following the manufacturer’s instructions (Invitrogen Corp., Carlsbad, CA). RNA quantity and quality was measured using NanoDrop 1000. After micro-cDNA synthesis (qScript cDNA Supermix, Quanta Biosciences), qPCR was performed using CFX96 Touch^TM^ detection system (Bio-Rad, Hercules, CA). The miRs primers miR-3613-3p and RNU-6 were purchased from Qiagen and 5 s rRNA was provided by Integrated DNA Technologies (Coralville, IA). The fold change of miR-3613-3p expression was calculated regarding U-6 and 5 s rRNA.

### Western blot

Western blot analysis was performed as previously described^44^. Briefly, protein concentrations were measured by Bradford method and 30 µg protein lysate was uploaded onto a 4–20% SDS-PAGE gel (Bio-Rad, Hercules, CA) in reducing conditions. After protein fractionation step, proteins were transferred on a nitrocellulose membrane followed by blocking in 5% BSA for 1 hour. The blocked membranes were incubated overnight at 4 °C with anti-CDC7 and anti-GAPDH (Santa Cruz Biotechnology, Dallas, TX), anti-phospho-ERK (T202/Y204) and anti-ERK1/2 (Cell signaling Tech., Danvers, MA), and phospho-MCM2 and anti-MCM2 (Bethyl, Montgomery, TX) antibodies. After serial washing and incubating the membranes with the appropriate secondary antibodies for 1 hour at room temperature, the signal was developed by either Amersham ECL Prime WB Detection Reagent (GE Healthcare Life Sciences, Pittsburgh, PA) or by incubation of membranes with fluorescent secondary antibody using Odyssey CLX Imaging System (Li-COR Biosciences, Lincoln, NE).

### Immunohistochemistry (IHC)

Prior of conducting any human-related studies, informed consent was obtained from all patients included in this study after IRB approval (IRB#2017-0190M) by Texas A&M University, College Station, Texas. Tissue microarray slide comprised 45 melanoma cases with 90 cores in addition to 10 normal skin cores was purchased from US Biomax, Inc. (Cat#ME1002a, Derwood, MD). The available clinical information was provided in Supplementary Table 1. IHC analysis with anti-CDC7 antibody (GeneTex Inc., Irvine, CA) was performed as we previously described^45^. Briefly, melanoma tissue sections and tissue microarray slide were de-waxed in two series of xylene and rehydrated in a descending series of ethanol. Melanoma tissue slides were then heated in 0.01 M citrate buffer (pH 6.0) for 20 min using steam cooker. The tissue sections were then immersed in 3% H_2_O_2_ for 10 min to block any endogenous peroxidase activity. These sections were incubated with anti-CDC7 antibody (Santa Cruz Biotechnology, Dallas, TX) overnight at 4 °C. The antigen-antibody complex was detected by ABC Elite Kit (Vector Laboratories, Burlingame, CA) and using 3, 3′-diaminobenzidine (DAB) as chromogen. Melanoma tissue sections were counterstained with Mayer’s hematoxylin solution (Newcomer Supply, Maddison, WI). The developed signals were captured and documented by Eclipse-80i microscope (Nikon Instruments, Melville, NY). The intensity of the positive staining was blindly examined and the histoscore was calculated as previously described by our group^45^.

### Statistical analysis

Comparison between experimental and control groups were achieved using Mann-Whitney statistical *t*-test (GraphPad Software, Inc., La Jolla, CA). The generated data was considered significant at p-value of less than 0.05. For microarray analysis, we used a cut-off value of 1 for log2 fold change, which corresponds to a 2-fold gene expression change. Adjusted p-value of <0.05 and absolute value of log2 fold change greater than 1 are considered as a conservative criterion for selecting differentially expressed miRs.

## Supplementary information

Supplementary Information

## Data Availability

The generated data of the current study are available upon request.

## References

[1] Moelling K, Heimann B, Beimling P, Rapp UR, Sander T (1984). Serine- and threonine-specific protein kinase activities of purified gag-mil and gag-raf proteins. Nature.

[2] Holderfield M, Deuker MM, McCormick F, McMahon M, Targeting RAF (2014). kinases for cancer therapy: BRAF-mutated melanoma and beyond. Nat Rev Cancer.

[3] Davies H (2002). Mutations of the BRAF gene in human cancer. Nature.

[4] Garnett MJ, Marais R (2004). Guilty as charged: B-RAF is a human oncogene. Cancer Cell.

[5] Gentilcore G (2013). Effect of dabrafenib on melanoma cell lines harbouring the BRAF(V600D/R) mutations. BMC cancer.

[6] Wan PTC (2004). Mechanism of Activation of the RAF-ERK Signaling Pathway by Oncogenic Mutations of B-RAF. Cell.

[7] Karoulia Zoi, Gavathiotis Evripidis, Poulikakos Poulikos I. (2017). New perspectives for targeting RAF kinase in human cancer. Nature Reviews Cancer.

[8] Bollag G (2012). Vemurafenib: the first drug approved for BRAF-mutant cancer. Nature reviews. Drug discovery.

[9] Alcala AM, Flaherty KT (2012). BRAF inhibitors for the treatment of metastatic melanoma: clinical trials and mechanisms of resistance. Clin Cancer Res.

[10] Beaumont KA (2016). Cell Cycle Phase-Specific Drug Resistance as an Escape Mechanism of Melanoma Cells. J Invest Dermatol.

[11] Inamdar GS, Madhunapantula SV, Robertson GP (2010). Targeting the MAPK pathway in melanoma: why some approaches succeed and other fail. Biochem Pharmacol.

[12] Griffin M (2017). BRAF inhibitors: resistance and the promise of combination treatments for melanoma. Oncotarget.

[13] Manzano JL (2016). Resistant mechanisms to BRAF inhibitors in melanoma. Annals of translational medicine.

[14] Sun X (2016). miR-7 reverses the resistance to BRAFi in melanoma by targeting EGFR/IGF-1R/CRAF and inhibiting the MAPK and PI3K/AKT signaling pathways. Oncotarget.

[15] Ji Z, Flaherty KT, Tsao H (2012). Targeting the RAS pathway in melanoma. Trends in molecular medicine.

[16] Montagnoli A., Moll J., Colotta F. (2010). Targeting Cell Division Cycle 7 Kinase: A New Approach for Cancer Therapy. Clinical Cancer Research.

[17] Masai H (2000). Human Cdc7-related kinase complex. *In vitro* phosphorylation of MCM by concerted actions of Cdks and Cdc7 and that of a criticial threonine residue of Cdc7 bY Cdks. J Biol Chem.

[18] Clarke LE (2009). Cdc7 expression in melanomas, Spitz tumors and melanocytic nevi. J Cutan Pathol.

[19] Shimizu T (2018). First-in-human phase 1 study of TAK-931, an oral cell division cycle 7 (CDC7) inhibitor, in patients (pts) with advanced solid tumors. Journal of Clinical Oncology.

[20] Namee NM, O’Driscoll L (2018). Extracellular vesicles and anti-cancer drug resistance. Biochim Biophys Acta Rev Cancer.

[21] Kaplan FM, Shao Y, Mayberry MM, Aplin AE (2011). Hyperactivation of MEK-ERK1/2 signaling and resistance to apoptosis induced by the oncogenic B-RAF inhibitor, PLX4720, in mutant N-RAS melanoma cells. Oncogene.

[22] Kaplan FM (2012). SHOC2 and CRAF mediate ERK1/2 reactivation in mutant NRAS-mediated resistance to RAF inhibitor. J Biol Chem.

[23] Le K, Blomain ES, Rodeck U, Aplin AE (2013). Selective RAF inhibitor impairs ERK1/2 phosphorylation and growth in mutant NRAS, vemurafenib-resistant melanoma cells. Pigment cell & melanoma research.

[24] Fedorenko IV, Gibney GT, Smalley KSM (2013). NRAS mutant melanoma: biological behavior and future strategies for therapeutic management. Oncogene.

[25] Kim JH, Ahn JH, Lee M (2017). Upregulation of MicroRNA-1246 Is Associated with BRAF Inhibitor Resistance in Melanoma Cells with Mutant BRAF. Cancer Res Treat.

[26] Chong GO (2015). Differential MicroRNA Expression Profiles in Primary and Recurrent Epithelial Ovarian Cancer. Anticancer research.

[27] Zhong S (2016). MicroRNA expression profiles of drug-resistance breast cancer cells and their exosomes. Oncotarget.

[28] Cao JX (2014). MiR-630 inhibits proliferation by targeting CDC7 kinase, but maintains the apoptotic balance by targeting multiple modulators in human lung cancer A549 cells. Cell death & disease.

[29] Bruck I, Kaplan D (2009). Dbf4-Cdc7 phosphorylation of Mcm2 is required for cell growth. The Journal of biological chemistry.

[30] Sasi NK, Bhutkar A, Lanning NJ, MacKeigan JP, Weinreich M (2017). DDK Promotes Tumor Chemoresistance and Survival via Multiple Pathways. Neoplasia.

[31] Jeggo PA, Pearl LH, Carr AM (2015). DNA repair, genome stability and cancer: a historical perspective. Nature Reviews Cancer.

[32] Nambiar S (2007). Identification and functional characterization of ASK/Dbf4, a novel cell survival gene in cutaneous melanoma with prognostic relevance. Carcinogenesis.

[33] Li X (2018). Nuclear PGK1 Alleviates ADP-Dependent Inhibition of CDC7 to Promote DNA Replication. Mol Cell.

[34] Jin S (2018). Cell division cycle 7 is a potential therapeutic target in oral squamous cell carcinoma and is regulated by E2F1. J Mol Med (Berl).

[35] Bhatt KV, Hu R, Spofford LS, Aplin AE (2007). Mutant B-RAF signaling and cyclin D1 regulate Cks1/S-phase kinase-associated protein 2-mediated degradation of p27Kip1 in human melanoma cells. Oncogene.

[36] Lopez-Bergami P, Fitchman B, Ronai Z (2008). Understanding signaling cascades in melanoma. Photochemistry and photobiology.

[37] Labib KH (2010). do Cdc7 and cyclin-dependent kinases trigger the initiation of chromosome replication in eukaryotic cells?. Genes Dev.

[38] Toress-Collado AX, Nazarian R, Jazirehi AR (2017). Rescue of cell cycle progression in BRAF(V600E) inhibitor-resistant human melanoma by a chromatin modifier. Tumour biology: the journal of the International Society for Oncodevelopmental Biology and Medicine.

[39] Søndergaard JN (2010). Differential sensitivity of melanoma cell lines with BRAFV600E mutation to the specific Raf inhibitor PLX4032. Journal of translational medicine.

[40] Montagnoli A, Moll J, Colotta F (2010). Targeting cell division cycle 7 kinase: a new approach for cancer therapy. Clinical cancer research: an official journal of the American Association for Cancer Research.

[41] Iwai K, Tadahiro N, Kurasawa O, Uchiyama N, Ohashi A (2016). A novel CDC7-selective inhibitor TAK-931 with potent antitumor activity. European journal of cancer.

[42] Munoz-Couselo E, Adelantado EZ, Ortiz C, Garcia JS, Perez-Garcia J (2017). NRAS-mutant melanoma: current challenges and future prospect. OncoTargets and therapy.

[43] Abd Elmageed ZY (2014). Neoplastic reprogramming of patient-derived adipose stem cells by prostate cancer cell-associated exosomes. Stem Cells.

[44] Abd Elmageed ZY (2018). Prognostic Role of BRAF(V600E) Cellular Localization in Melanoma. J Am Coll Surg.

[45] Abd Elmageed ZY (2017). Immunohistochemistry as an accurate tool for evaluating BRAF-V600E mutation in 130 samples of papillary thyroid cancer. Surgery.

