# Supplementary material for: Targeting CDC7 sensitizes resistance melanoma cells to BRAFV600E-specific inhibitor by blocking the CDC7/MCM2-7 pathway
Source: Sci Rep. 2019 Oct 2;9:14197. doi: 10.1038/s41598-019-50732-w (PMC6775054; doi:10.1038/s41598-019-50732-w)

Targeting CDC7 sensitizes resistance melanoma cells to BRAF^{V600E}-specific inhibitor by blocking the CDC7/MCM2-7 pathway

Shaimaa A. Gad[1,4], Hamdy EA Ali[1], Rofaida Gaballa[1], Rania M. Abdelsalam[2], Mourad Zerfaoui[3], Hamed I. Ali[1], Salwa H. Salama[4], Sanaa A. Kenawy[2], Emad Kandil[3], Zakaria Y. Abd Elmageed[1]

[1]Department of Pharmaceutical Sciences, Rangel College of Pharmacy, Texas A&M Health Science Center, Kingsville, TX 78363, [2]Department of Pharmacology and Toxicology, Faculty of Pharmacy, Cairo University, [3]Department of Surgery, Tulane University School of Medicine, New Orleans, LA 70118, [4]Department of Pharmacology, Medical Division, National Research Center, Cairo.

Supplementary Information

Supplementary Table 1. Clinical information of melanoma patients used in the study

**Parameter**	**Description**	**Mean (%)**
Age (Mean±SD)		54.4 ± 14.2
Gender	Male	50 (55%)
	Female	40 (45%)
Organ	Skin	70 (77.7)
	Rectum	10 (11.1)
	Vulva	6 (6.6)
	Others	4 (4.4)
T-stage	T1+2	10 (11.1)
	T3+4	70 (77.7)
Pathological stage	I	6 (6.6)
	II	62 (68.9)
	III+IV	12 (13.3)

Supplementary Table 2. Correlation of CDC7 expression with clinical outcomes of melanoma patients

Parameter		Cyto.	Nucl.	Age	Gender	TNM	Stage
Cyto.	*r*	1.0000	0.0048	0.3195	0.2547	0.1463	0.2810
	p		0.9656	**0.0034**	**0.0209**	0.2200	**0.0167**
Nucl.	*r*		1.0000	-0.0623	-0.0764	0.1869	0.1016
	p			0.5777	0.4948	0.1157	**0.0167**
Age	*r*			1.0000	-0.0103	0.1498	0.0735
	p				0.9229	0.1845	0.5224
Gender	*r*				1.0000	-0.1744	0.2059
	p					0.1216	0.0704
TNM	*r*					1.0000	0.6348
	p						0.0004
Stage	*r*						1.0000
	p						

Correlation coefficient (*r*) was calculated among different groups and significant data considered at $p<0.05$ (bold numbers).

Supplementary Figure 1. ***Representative photomicrograph showing the effect of Vemurafenib treatment on parental (P) and Vemurafenib resistant (R) melanoma cells.*** Total cell lysate collected at different time points (from 1-24h) of 2.5 µM Vemurafenib treatment. Western blot analysis was performed and membranes were probed with the designated antibodies as shown. B-actin was used as a loading control. Protein signals were detected by Odyssey CLX Imaging System. M: protein marker

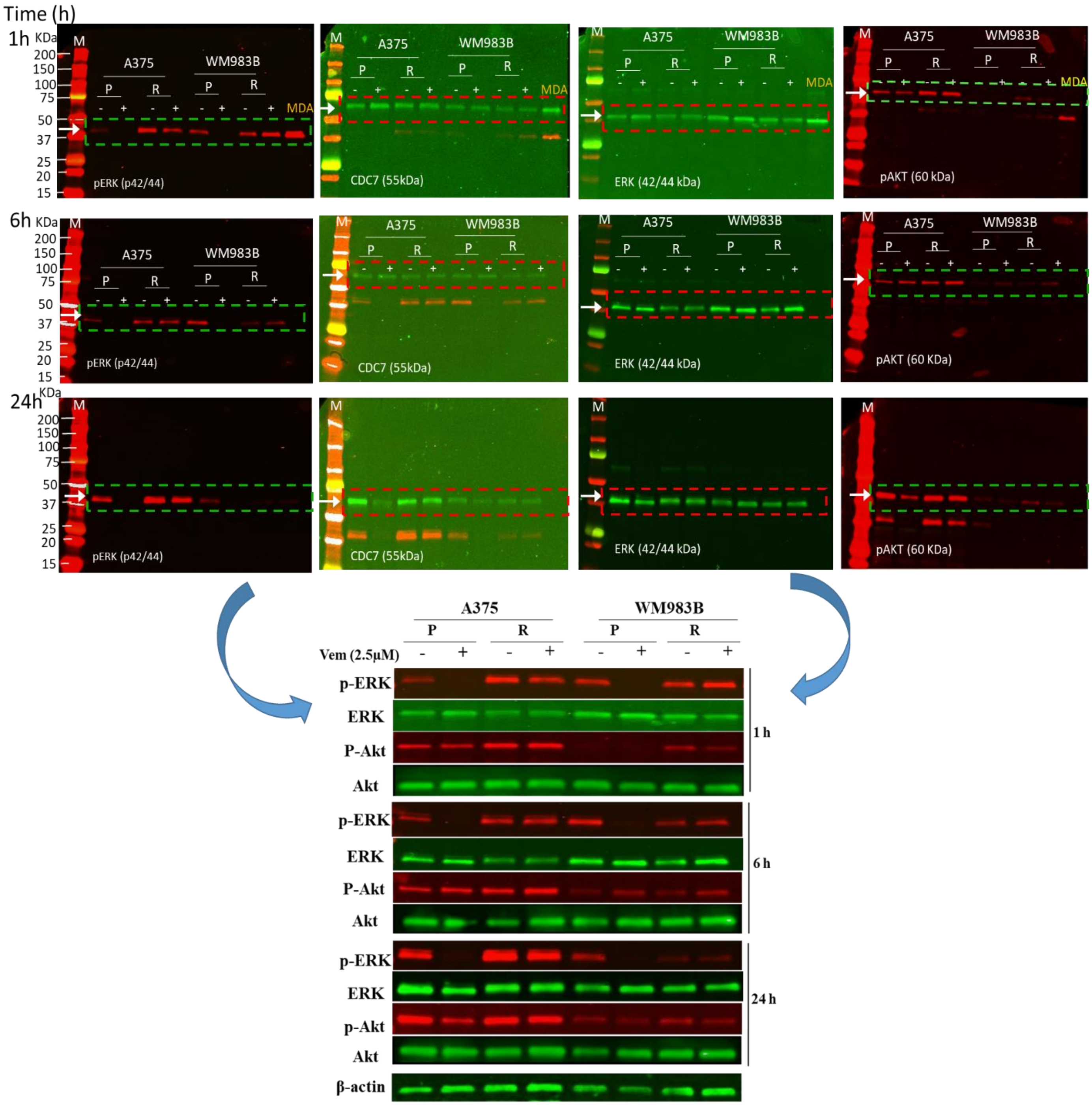

Supplementary Figure 2. ***Representative photomicrograph of endogenous expression of CDC7 in sensitive (P) and resistant (R) cells before and after treatment with Vemurafenib.*** Total cell lysate collected before (A) or after (B) treatment of melanoma cells with 2.5µ Vemurafenib at different time points (24 & 48h). Western blot analysis was performed and membranes were probed with the designated antibodies. GAPDH or β-actin was used as loading control.
M: protein marker

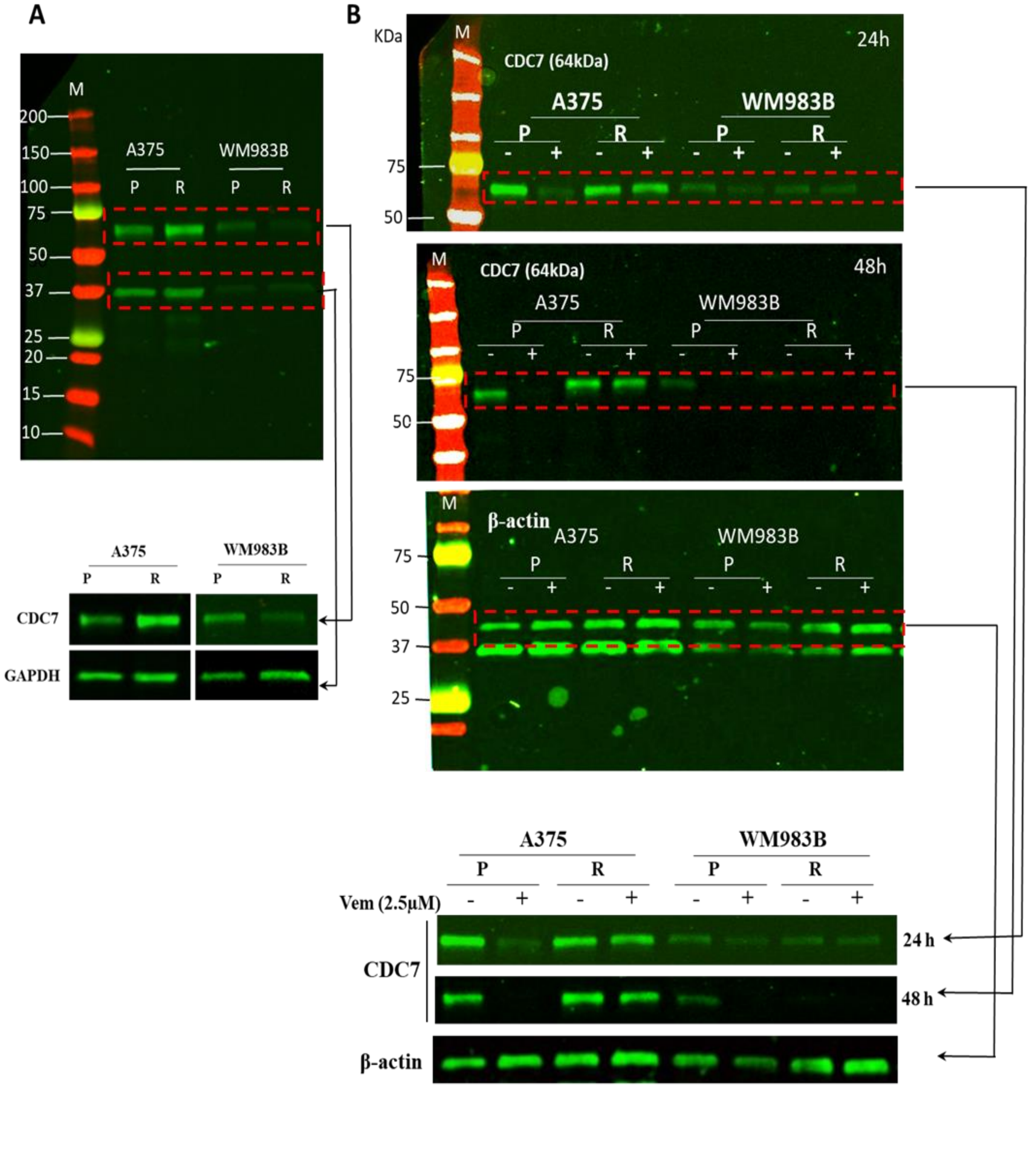

Supplementary Figure 3. ***Representative photomicrograph showing targeting of CDC7 and phosphorylated ERK1/2 by miR-3613 mimic.*** Total cell lysate collected after transfection of A375 cells with 25 & 50nM miR-3613-3p mimic in addition control cells transfected with non-specific siRNA (scrambled). Western blot analysis was performed and membranes were probed with the designated antibodies. GAPDH was used as a loading control.

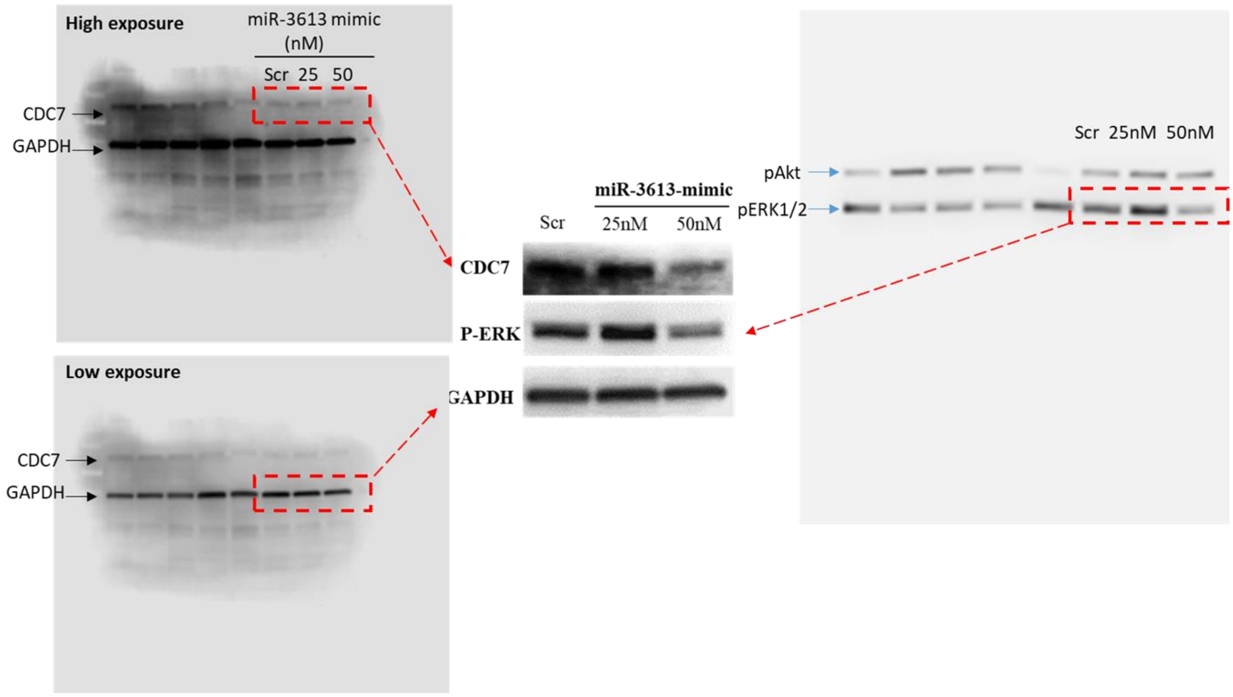

Supplementary Figure 4. ***Representative photomicrograph after treatment of A375-R cells with Vemurafenib or CDC7 inhibitor TAK-931.*** Total cell lysate collected after treatment of A375-R cells with either 2.5µ Vemurafenib or 500nM or 1000nM TAK-931 at different time points. Western blot analysis was performed and membranes were probed with the designated antibodies.

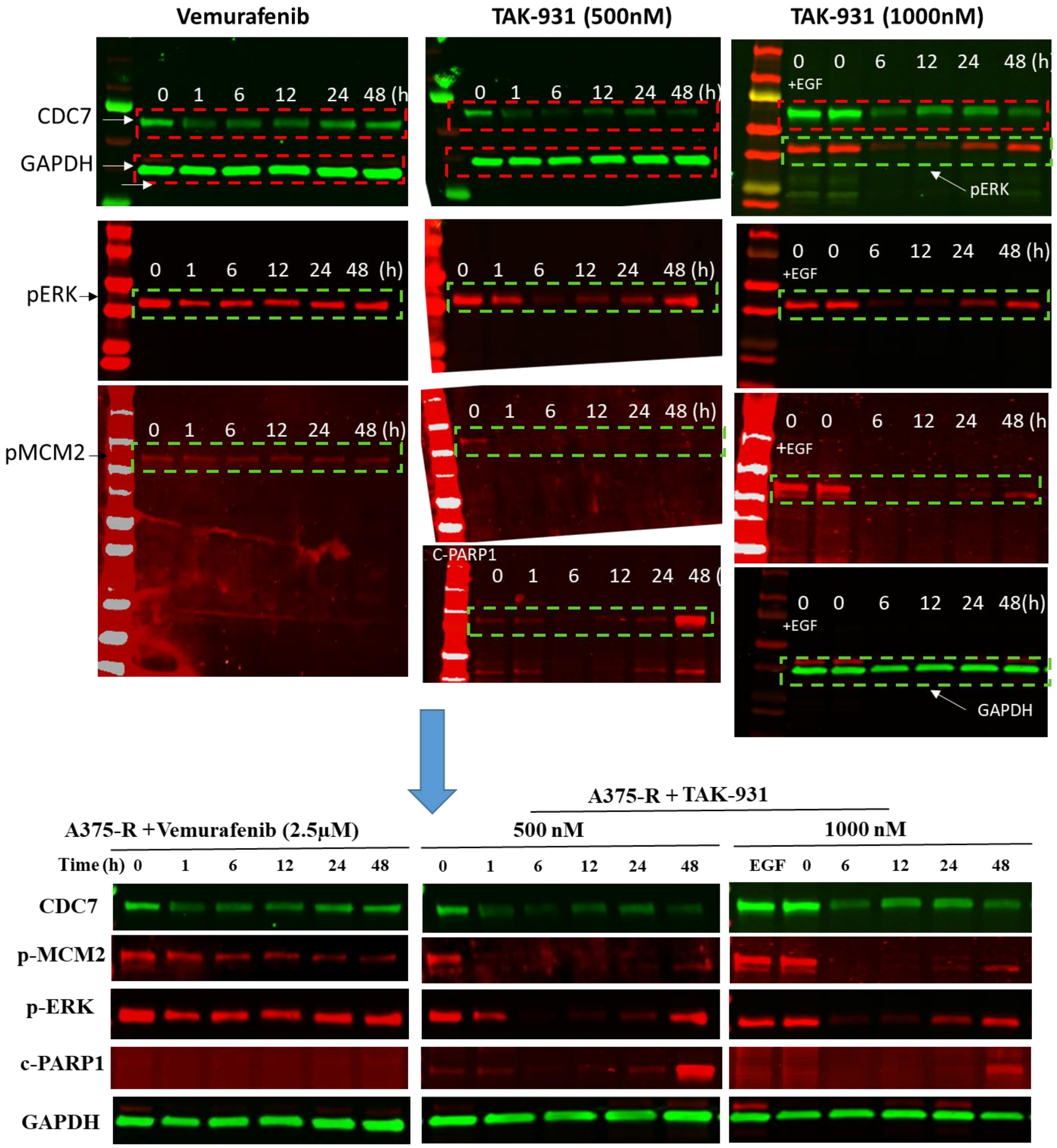

Supplement: Supplementary file 1 — Supplementary Information [file 41598_2019_50732_MOESM1_ESM.pdf]
